# Supplementary material for: Verification of the Four-Stage Model of Humor Processing: Evidence from an fMRI Study by Three-Element Verbal Jokes
Source: Brain Sci. 2023 Feb 27;13(3):417. doi: 10.3390/brainsci13030417 (PMC10046724; doi:10.3390/brainsci13030417)
Supplement: Supplementary file 1 [file brainsci-13-00417-s001.zip › brainsci-2152330-supplementary.pdf]

Supplementary Material

Verification of the Four-Stage model of Humor Processing: Evidence from an fMRI Study by Three-Element Verbal Jokes

Chia-Yueh Chang[1], Yu-Chen Chan[2], Hsueh-Chih Chen[1, 3,4,5,*]

[1] Department of Educational Psychology and Counseling, National Taiwan Normal University, Taipei, Taiwan. rainbowchang0226@gmail.com (C.-Y.C.)

[2] Department of Educational Psychology and Counseling, National Tsing Hua University, Hsinchu, Taiwan. ycchan@mx.nthu.edu.tw (Y.-C.C.).

[3] Institute for Research Excellence in Learning Sciences, National Taiwan Normal University, Taipei, Taiwan.

[4] Chinese Language and Technology Center, National Taiwan Normal University, Taipei, Taiwan.

[5] NSTC AI Biomedical Research Center, Tainan, Taiwan.

[*] Correspondence:

Hsueh-Chih Chen

E-mail: chcjyh@gmail.com; TEL: (886) 2-7734-3782; FAX: (886) 2-2341-3865

Supplementary Material

Table S1-1, S2-1, S3-1, and S4-1 offer 4 examples of the three-element stimuli used in the study, showing three elements (setup, first punch line, and second punch line) correspond to four humor processes (expectation, incongruity, resolution, and elaboration). The examples are given in the original Mandarin Chinese, showing the matches for length and punctuation across the joke and nonjoke conditions. Table S1-2, S2-2, S3-2, and S4-2 provide English translations.

Table S1-1. Sample 1 stimuli in Mandarin Chinese.

<table>
<tr><td rowspan="2"></td><td colspan="3">Humor element
(Humor processing)</td></tr>
<tr><td>Setup
(Expectation)</td><td>First punch line
(Incongruity)</td><td>Second punch line
(Resolution, Elaboration)</td></tr>
<tr><td>Joke</td><td rowspan="2">精神病院院長：「明天總統來，你們都要拍手，表現好一人一肉包。」隔天病人果然狂拍。</td><td>突然，一個病人衝出去，伸手打了總統一個巴掌，</td><td>凶狠狠地說：「你不想吃肉包嗎？」</td></tr>
<tr><td>Nonjoke</td><td>到了晚上，院長依約按數量準備肉包，每個人都拿到了大肉包。</td><td>大家吃得津津有味，且一致認為這是份好差事。</td></tr>
</table>

Table S1-2 English translations of the sample stimuli 1.

<table>
<tr><td rowspan="2"></td><td colspan="3">Humor element (Humor processing)</td></tr>
<tr><td>Setup
(Expectation)</td><td>First punch line
(Incongruity)</td><td>Second punch line
(Resolution, Elaboration)</td></tr>
<tr><td>Joke</td><td>The director of the psychiatric hospital: “Tomorrow the president will come,</td><td>Suddenly, a patient rushed out, reached out and slapped the president in the face.</td><td>“You don't want to eat meat buns?” The patient said fiercely.</td></tr>
</table>

Nonjoke	you all have to clap your hands, and if you do well, you will get a meat bun." The next day, the patients did clap wildly.	At the end of the day, the director prepared the meat buns according to the quantity promised, and everyone got a big meat bun.	They all enjoyed the food and agreed that it was a good job.

Table S2-1. Sample 2 stimuli in Mandarin Chinese.

	Humor element (Humor processing)		
	Setup (Expectation)	First punch line (Incongruity)	Second punch line (Resolution, Elaboration)
Joke	小明在學校跟小志打架，帶了黑眼圈回家。媽媽：「明天帶塊蛋糕給小志，跟他和好。」	第二天下課，媽媽發現小明帶了更大的黑眼圈從學校回來。	小明說：「小志說他還想吃蛋糕。」
Nonjoke		第二天下課，小明臉上開心地掛著笑容、平安無事地回到家。	小明：「多虧了蛋糕，我已經與小志和好了！」

Table S2-2 English translations of the sample stimuli 2.

	Humor element (Humor processing)		
	Setup (Expectation)	First punch line (Incongruity)	Second punch line (Resolution, Elaboration)
Joke	Peter fought with Jack at school and came home with a black eye.<br>Mom: "Tomorrow, bring a cake to Jack and make up with him."	The next day after class, mother found that Peter had come back from school with even bigger dark circles under his eyes.	Peter said, "Jack said he still wants to eat cake."
Nonjoke		The next day after class, Peter returned home with a happy smile on	Peter: "Thanks to the cake, I've made up with Peter!"

		his face and safe and sound.	

Table S3-1. Sample 3 stimuli in Mandarin Chinese.

<table>
<tr><td rowspan="2"></td><td colspan="3">Humor element
(Humor processing)</td></tr>
<tr><td>Setup
(Expectation)</td><td>First punch line
(Incongruity)</td><td>Second punch line
(Resolution, Elaboration)</td></tr>
<tr><td>Joke</td><td rowspan="2">小志在辦公室老是放響屁，又吵又臭。同事忍不住婉轉地說：「你能不能不出聲？」</td><td>說完便見小志坐在那搖來晃去, 身體上下左右抖個不停。</td><td>小志解釋道：「我調成振動的了！」</td></tr>
<tr><td>Nonjoke</td><td>小志不好意思地試了幾次之後都無法控制聲音，索性走出辦公室。</td><td>小志自言自語：「這樣就不會吵到同事們了。」</td></tr>
</table>

Table S3-2 English translations of the sample stimuli 3.

	Humor element (Humor processing)		
	Setup (Expectation)	First punch line (Incongruity)	Second punch line (Resolution, Elaboration)

Joke	Jack always farted loudly in the office, and it was noisy and smelly. A colleague couldn't help but politely say, "Can you keep your voice down?	After saying that, Jack sitting there shaking around, his body shaking up and down.	Jack explained, “I've adjusted it to vibrate!”
Nonjoke		Jack was embarrassed that he couldn't control his voice after a few attempts, so he simply walked out of the office.	Jack said to himself, “This way, I won't disturb our colleagues.”

Table S4-1. Sample 4 stimuli in Mandarin Chinese.

	Humor element (Humor processing)		
	Setup (Expectation)	First punch line (Incongruity)	Second punch line (Resolution, Elaboration)
Joke	婦女帶著女兒去看精神科。婦女：「我女兒這半年來，一直覺得自己是一隻母雞。」	醫生:「都已經半年了,為什麼到現在才來看病呢？」	婦女：「因為我們一家人都在等著吃雞蛋！」
Nonjoke		醫生：「我開一些藥物給你們拿回去，讓妳女	婦女感激涕零的說：「好的，謝謝醫生！」

		兒按時吃, 然後下周回診。」	

Table S4-2 English translations of the sample stimuli 4.

	Humor element (Humor processing)		
	Setup (Expectation)	First punch line (Incongruity)	Second punch line (Resolution, Elaboration)
Joke	A woman takes her daughter to visit the psychiatric department. Woman: My daughter has always thought that she is a hen for the past 6 months.	Doctor: It's been 6 months, why have you come to see me now?	Woman: Because my family is waiting to eat eggs!
Nonjoke		Doctor: I will prescribe some drugs for your daughter, have her take them on time, and then come back next week,	Woman (said with gratitude): Thank you doctor!
